# Practice makes perfect, but to what end? Computerised brain training has limited cognitive benefits in healthy ageing

**DOI:** 10.1007/s00426-025-02110-7

**Published:** 2025-03-24

**Authors:** Emma Sutton, Jonathan Catling, Jet J. C. S. Veldhuijzen van Zanten, Katrien Segaert

**Affiliations:** 1 School of Psychology, College of Life and Environmental Sciences, University of Birmingham, Birmingham, UK; 2 Centre for Developmental Science, School of Psychology, College of Life and Environmental Sciences, University of Birmingham, Birmingham, UK; 3 School of Sport, Exercise, and Rehabilitation Sciences, College of Life and Environmental Sciences, University of Birmingham, Birmingham, UK; 4 Centre for Human Brain Health, School of Psychology, College of Life and Environmental Sciences, University of Birmingham, Birmingham, UK

## Abstract

Whether brain training programmes are effective and have transferable benefits to wider cognitive abilities is controversial, especially in older adult populations. We assessed, in a randomised controlled intervention study, whether a commercially available brain training programme can induce cognitive improvements in a sample of healthy older adults (*N* = 103). Participants completed a three-month intervention of either an adaptive computerised cognitive training programme (through a brain training app) or active control. Cognition was measured through a comprehensive battery of tasks pre- and post-intervention to assess working memory, processing speed, attention, and language functioning. Participants in the intervention group significantly improved on all tasks that were trained specifically within the brain training programme (i.e. practice effects). However, for the cognitive tasks assessed pre- and post-intervention there was no evidence of any of these practice effects transferring to improvements in cognitive outcome measures compared to the active control group (i.e. transfer effects). Our results indicate that the benefits of brain training programmes appear to be limited to practice effects of trained tasks, while no evidence is found for transfer effects to other, related or unrelated, untrained cognitive tasks.

## Introduction

Cognitive processes such as working memory, processing speed, attention, and language functioning all decline during healthy ageing (Reuter-Lorenz et al., 2021; Salthouse, 2010; Segaert et al., 2018). As life expectancy in developed countries continues to increase (Roser et al., 2013), mitigating age-related cognitive decline has become an increasingly popular field of research. In the present study, we examined whether computerised cognitive training improves performance across multiple domains of cognition.

A recently popular method of delivering cognitive training has been to use commercially available brain training programmes. Applications such as Lumosity (Lumosity, 2023), Peak (Peak, 2023) and BrainHQ (BrainHQ, 2023) are commercially advertised as training programmes that will improve cognitive ability and delay cognitive decline. These applications are easy to use, relatively affordable, adaptive (increasing in difficulty with improved performance, which is key for cognitive training programmes to work (Brehmer et al., 2012) and include training games that cover a variety of cognitive processes such as short-term memory, language, attention, and processing speed.

Support for the effectiveness of brain training programmes in healthy older populations is mixed. There is evidence from reviews and meta-analyses that computerised cognitive training or brain training leads to small but significant improvement in skills such as working memory, processing speed, and visuospatial skills in healthy older adults (Bonnechere et al., 2020; Kueider et al., 2012; Tetlow & Edwards, 2017). Conversely, papers report that efficacy varies across cognitive domains and can be affected by design choices (Lampit et al., 2014), and a recent meta-analysis has found no convincing improvement after accounting for publication bias (Nguyen et al., 2022). Older adults have higher expectations of brain training compared to younger adults (Rabipour & Davidson, 2015), and they could arguably benefit most from their use, if effective. Whether brain training programmes lead to tangible improvements in cognitive abilities in healthy older adults therefore warrants further investigation.

Some of the inconsistencies found in cognitive training research more broadly can be attributed to methodological differences (Green et al., 2014; Noack et al., 2014; Simons et al., 2016). Sample sizes vary substantially and are often limited; 50% of studies in a 2014 review of transfer effects in cognitive training studies had fewer than 20 participants in each group (Noack et al., 2014), and 90% had fewer than 45 in each group. Training duration is also often limited; 50% of studies reported 8 h and 20 min of training or less, with the majority (90%) reporting less than 20 h in total (Noack et al., 2014). Another concern is the size and content of the test battery (Green et al., 2014). Many studies, especially early studies when cognitive training was in its infancy, used a small test battery (i.e., one test per cognitive function) to assess cognitive outcome measures. However, to assess valid training benefits, the outcome measures need to be chosen such that they assess changes across the construct rather than the individual tasks. For example, executive function would ideally not be assessed by a single measure: executive function itself is made up of smaller subprocesses (inhibition, shifting and updating (Sandberg et al., 2014), so one outcome measure that focuses on one of those processes is not enough to encompass executive function as a whole. Moreover, if cognitive training includes a specific task that trains, for example, working memory (e.g., an n-back task), then it’s true benefits can only be assessed through performance on a different task which measures skills within this domain (i.e. a task which also assesses working memory but is not an n-back task) to rule out that improvements are mere practice effects. A final consideration is the choice of control group (Simons et al., 2016). The gold standard is to use an active control group that mimics the intervention as closely as possible, while leaving out the ‘active ingredient’ of the training. However, the very nature of cognitive training programmes makes this difficult. The type of control groups in published studies therefore varies, often including passive control groups, and not always accounting for placebo effects, motivation, or cognitive demands (Simons et al., 2016). Active control groups can be divided further; into ‘active-ingredient’ controls and ‘similar-form’ controls (Masurovsky, 2020). ‘Active-ingredient’ control groups are identical in every aspect apart from the ‘active’ ingredient, but these are difficult to implement and in practice are rarely used. For example, Brehmer et al. (2012) tested whether adaptivity of training was key to improvements in working memory. The control group training was identical to the intervention, but the difficulty remained the same throughout. The ‘active ingredient’, and the only thing that changed, was adaptable difficulty. ‘Similar-form’ active controls are much more common, mimicking aspects of the training but differing in more than one way, such as comparing computerised cognitive training to video games that are not designed to train cognitive domains (Ballesteros et al., 2017). ‘Similar-form’ control groups are still considerably more suitable than passive or no-contact control groups (Masurovsky, 2020).

We note that in the above set of issues, a key concern, but one most often overlooked, is the need to establish evidence of *transfer* effects (the benefits of the training ‘transferring’ to other, untrained, cognitive tasks), as opposed to practice effects (improvements within the training, or same tasks, itself). Transfer effects can be categorised by how similar they are to the trained cognitive domain (Sala et al., 2019). Near transfer refers to skills generalising to similar domains (e.g., training in working memory which is transferring to other, related but untrained, working memory tasks), while far transfer relies on the cognitive domain being weakly related, or not related at all, to the trained domain (e.g., working memory training which is transferring to language, or executive control benefits; (Sala et al., 2019). The more shared features there are between domains, the nearer the transfer effects (Sala et al., 2019). Of course, the ultimate aim of brain training programmes is that training of specific cognitive processes leads to improvements across cognitive domains (Stojanoski et al., 2018). There is some evidence that brain training can lead to transfer effects (McDougall & House, 2012), however, there are also cases where no transfer benefits are found at all (Kable et al., 2017; Stojanoski et al., 2018). Even when papers report significant positive effects of brain training programmes on cognition in healthy older populations, the effects are often driven by improvements on very near transfer tasks (Lee et al., 2020), and little to no evidence of far transfer is established. Furthermore, a recent meta-analysis of brain training randomised controlled trials with older adults found small but significant transfer to some cognitive domains, however most effects were no longer significant once publication bias was taken into account (Nguyen et al., 2022). There are also cases where previously reported effects have perhaps been exaggerated. Brain training research sometimes describes improvements in trained effects (improvement in performance within the programme) and report these as an improvement in cognitive ability (Bonnechere et al., 2021). Instead, these are in fact practice effects and do not necessarily entail improvements in cognitive function, since transfer effects (near or far) were not established or were not even assessed. Transfer effects are essential if a training programme is going to be effective and wide-reaching, especially in ageing populations, but concrete evidence for them is often lacking.

Due to these inconsistencies and the controversy surrounding brain training programmes and their effects, there is a need for robust and rigorous research to assess its efficacy. An extensive review paper has given recommendations for how research into brain training programmes should be conducted and published (Simons et al., 2016). The researchers recommended a large sample size with random allocation to groups and blinding of conditions if possible. An appropriate active control group should be utilised, meaning a control group that correctly mimics the level of engagement of the intervention, but that theoretically will not result in improved cognitive performance. This allows for placebo effects to be controlled for, and any effects to be attributed to the ‘active’ ingredient of the training programme (Simons et al., 2016). Furthermore, interventions need to control for expectations and motivations of both groups. Finally, the researchers recommend using appropriate outcome measures and a test battery using multiple tasks to measure each construct. Our study has incorporated each of these key recommendations.

To assess the possible cognitive benefits of the training we measured cognition across a wide range of domains. Among various possible cognitive functions of interest, working memory stands out as a commonly reported function. This is not only due to its consistent decline with age (Salthouse, 2010) but also because it serves as a foundation for many other cognitive abilities. Working memory training has shown convincing improvements in memory skills in older adults in recent years (Karbach & Verhaeghen, 2014). Another cognitive skill that exhibits consistent decline with age is processing speed, which has been effectively trained in older adults: the well-known ACTIVE study demonstrated significant and sustained improvements in processing speed over a two-year (Ball et al., 2002) and ten-year (Rebok et al., 2014) period. Although findings on attention skills are not always consistent, attention skills do undergo changes with age (Veríssimo et al., 2022), and deficits in attention can impact daily life (Glisky, 2007), making it a worthwhile line of enquiry. Finally, language problems, specifically word finding difficulties, increase with age (Maylor, 1990) (Segaert et al., 2018) and are commonly reported as noticeable deficits by older adults. Assessments of language function are not often included in brain training research, but due to the relevance of language abilities to ageing, we believed it would be interesting to include a language assessment in the present study.

In sum, cognitive training is an important field of research that needs methodologically sound experiments to assess whether brain training programmes are effective in healthy older adult populations. The aim of the current study was to do just that: to assess the efficacy of a commercially available adaptive brain training programme (Peak) for improving function in a range of cognitive domains, using a randomised controlled study with healthy older adults.

Popular brain training applications including Lumosity, BrainHQ, Elevate, and Peak have many similarities; they aim to improve cognition more broadly by training specific cognitive tasks in domains like attention and memory. These applications use adaptive training, track user performance and scores over time, and some (including Peak) compare scores to other users or age groups. It can therefore be difficult to choose one over another. We chose Peak because it includes some games/exercises that also target emotional capacity and language skills (Lumosity and BrainHQ do not include this). Further, compared to its counterparts, it is relatively under-researched and reported on; to our knowledge, Peak has not been used as a cognitive training programme in a randomised controlled trial with an active control group.

We aimed to include a larger sample size than has been used in many previous cognitive training studies (Noack et al., 2014) and an appropriate active control group. We assessed cognitive functions known to decline with healthy ageing and used tasks that are commonly used in ageing research. These included working memory (Forward Digit Span task and visual N-back task), processing speed (Choice Reaction Time task and Letter Comparison task), attention (Attention Network Task) and language functioning (tip-of-the-tongue task). We hypothesised that we would find significant improvements within the training games (practice effects) for our intervention group. Whether we would find transfer effects from the brain training to other cognitive abilities was uncertain, though we anticipated any transfer effects would be to similar cognitive tasks (near transfer) rather than to dissimilar tasks (far transfer).

## Methods

### Participants

**Fig. 1:**
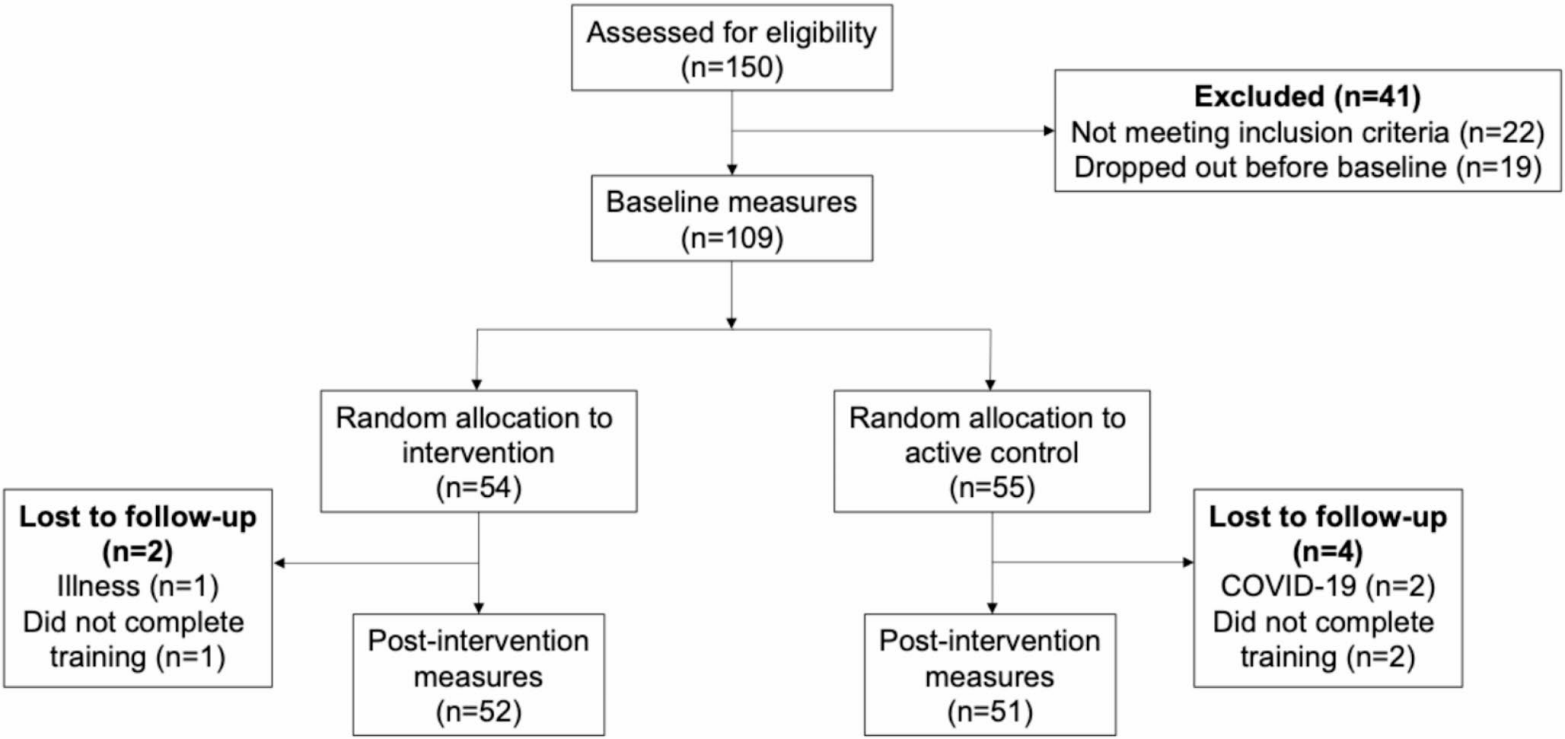
Participant flow chart

One hundred and nine participants were initially recruited through existing participant databases at the University of Birmingham (Birmingham 1000 Elders) and online social media advertisements. Six participants dropped out and did not complete the post-intervention measures; 103 completed both pre- and post-intervention sessions. Participants ranged from 60 to 84 years old (M = 70.57, SD = 5.5). The Montreal Cognitive Assessment (Nasreddine et al., 2005) was used to exclude participants with suspected impaired cognition. A recent meta-analysis suggested that a cut-off score of 23 out of 30 for mild cognitive impairment (rather than the original 26/30) provides fewer false positives and better diagnostic accuracy (Carson et al., 2018); no participants were excluded based on this cut-off point. A participant flow chart can be found in Fig. 1.

Inclusion criteria required participants to be over 60 years of age, a monolingual native English speaker, have normal or corrected-to-normal hearing and vision, access to a smartphone or tablet, and be able to walk short distances unaided. Participants were also required to be up to date on COVID-19 vaccinations at the time of visit. Exclusion criteria were as follows: a diagnosis of cognitive impairment (e.g., dementia, mild cognitive impairment), a diagnosis of mental health disorder (e.g., depression, anxiety), a diagnosis of language impairment (e.g., dyslexia). Participants who reported they were regularly taking medication known to interact with cognitive function or inflammation (e.g., medication for arthritis, anti-depressants) were also excluded.

### Intervention

#### Cognitive training intervention

The present study used Peak (Peak, 2023), a commercially available brain training application, as the intervention. A total of 48 games focus on seven different categories: Language, Problem Solving, Memory, Focus, Mental Agility, Emotion, and Coordination. The short games focus on abilities such as vocabulary, working memory, visual or sustained attention, mathematics, and task shifting. Some games within Peak closely resemble the outcome measures used in the present study. As such, our outcome measures allow the assessment of near-transfer as well as far-transfer. For example, Rush Back and Rush Back + are N-back style games, and so assessment of N-back performance would reflect near transfer. On the other hand, the language-based games within Peak tap into fluency and vocabulary, rather than phonological access (which is what tip-of-the-tongue problems are related to). The tip-of-the-tongue task would therefore reflect far transfer.

The app tracks progress, comparing one’s scores to personal previous scores and to other players of a similar age group. Scores are given out of a possible 1000 and are updated for each category every time a game or ‘brain workout’ is played. Games are adaptive, increasing in difficulty with improved performance, and are either timed (times ranging from 45 s to around 4 min) or are self-directed. Participants in our study’s intervention group were asked to use the pre-made ‘daily brain workouts’ which randomly selects six out of the 48 games each day to ensure a wide range of cognitive abilities were trained. The daily workouts took around 15 minutes, after which participants were told they could explore the app and use other games as they wished. Peak has been reported by others to improve everyday functioning and improve measures of processing speed in younger adults with depression (Motter et al., 2019), however to our knowledge, no research to date has investigated the efficacy of Peak on cognition in healthy ageing.

#### Active control group

Active control groups, matched to the intervention as best as possible on training intensity, motivation, and engagement, are the gold standard in intervention studies (Green & Bavelier, 2012). Active control groups are crucial to establish that any effects found are a direct result of the intervention itself, rather than social impacts, expectation effects of training, or the repetition or structure of completing a daily or regular task (Green et al., 2014). Following the justification of an active control group given by Kable et al. (2017), the control group was chosen to mimic and control for any effects of cognitive stimulation. The choice of training was a ‘similar-form’ control group (Masurovsky, 2020), a readily available free mobile application, Cardgames.io (Cardgames.io, 2023), that includes 42 card and board games, such as Hearts, Rummy, Yahtzee, and Solitaire. Similar to Kable et al. (2017), participants were not restricted in terms of what games they played but were encouraged to try a variety of them, as long as it was for at least 15 min per day, to match training intensity. Crucially, game difficulty was not adaptive to user performance, and the variety of games ensured novelty and engagement.

### Cognitive outcomes measured pre- and post-intervention

#### Working memory

Working memory was assessed through two computerised tasks: the Forward Digit Span task, and N-back task.

##### Forward digit span

Single digits (0–9) are presented to participants one at a time on a computer screen at 1000 ms intervals. Trials begin with three-digit sequences up to a possible ten-digit sequence. After the sequence has been presented, participants are asked to type the digits in the order they were presented using the computer keyboard, using the Enter key to confirm. Feedback is given after each trial. There are three trials in each level, and participants must get two trials correct to move up to the next level. The task automatically ends after two incorrect answers within a level. The participant’s digit span is taken from the highest level in which participants get two trials correct. Measuring working memory using Digit Span tests is common in psychological experiments such as these and has been established within the ageing literature (Grégoire & Van der Linden, 1997). The forward Digit Span is suggested to mostly tap into grouping and short-term retention (Feier & Gerstman, 1980). This task takes approximately 5 min.

*N-back task*: This task consists of a visual N-back task. Participants view a 3 × 3 grid on a computer screen, in which squares appear in one of the nine possible places (presented regularly at 1000 ms intervals), one at a time. Participants are asked to respond by pressing the ‘Space’ key whenever the target matches the location shown N steps before. There are two blocks of 60 trials, a 1-back and a 2-back, with 16 targets in the 1-back block, and 12 in the 2-back. Participants have a practice 1-back block of 12 trials and receive feedback on their performance at the end of the practice. No feedback is given for rest of the task. This task is well established and has been validated as an effective working memory measure (Jaeggi et al., 2010). RT is measured in milliseconds and a performance is measured by calculating a d’ score from the four outcomes of each trial: hit (correct button press in response to the target), miss (no button press when the target appears), correct rejection (participant correctly does not press button) and false alarm (participant incorrectly presses button when no target appears). The final score, d’, is calculated as follows:

Z-score Hit Rate [#Hits / (#Hits + #Misses)] minus Z-score False Alarm Rate [#False Alarms / (#False Alarms + #Correct Rejections)]

A higher score indicates better performance or better sensitivity to the target. The task takes approximately 10 min.

#### Processing speed

Processing speed was assessed through two computerised tasks, the Choice RT task, and Letter Comparison task.

##### Choice RT task (CRT)

Four empty squares are displayed on the computer screen, which correspond to the Z, X, N, and M keyboard keys. Participants rest index and middle fingers on these keys. Intertrial intervals ranged from 1000 and 2500ms (at 250ms increments), after which an X appears in one of the four squares. Participants are asked to press the Z, X, N, or M key for which box the X appears in, as fast and accurately as possible. The task consists of 8 practice trials, followed by 32 test trials. Feedback is only given after each practice trial. Target locations and intertrial intervals are counterbalanced, and trials are randomised. RT is calculated in milliseconds. This task is well established when assessing processing speed (Deary et al., 2010). The CRT takes approximately 2 min.

##### Letter comparison task (LC)

A fixation cross is displayed in the middle of the screen. Two sequences of letters are then displayed at equal distances above and below the fixation cross, and participants are asked to decide whether the letter sequences are the same or different. Participants are asked to do this as fast and accurate as possible. The Z arrow key is pressed for the same letters, M for different. The task consists of eight practice trials of three letter sequences, followed by one block of 48 trials, consisting of three-letter and six-letter strings. Trials are presented at even intervals (1000ms between trials, fixation cross for 500ms, blank for 100ms, letters presented until response) and are randomised. RT is calculated in milliseconds. This task is a well-known measure of processing speed in ageing populations (Salthouse & Babcock, 1991). The task takes approximately 5 min to complete.

#### Attention

*Attention Network Task (ANT)*: The Attention Network Task was used to measure attention. While being a single task for the participant, this computerised task allows assessment of the three key domains of the attention network - orienting, alerting and executive control - through the combination of a flanker (Eriksen & Eriksen, 1974) and spatial attention task (Posner & Cohen, 1984). During each trial, a fixation cross is displayed for 400ms before a stimulus appears. The stimulus is a row of five arrows, each pointing left or right. Participants are asked to report, using the left and right arrow keyboard keys, which direction the centre arrow points. They are asked to do this as fast and accurate as possible. The centre arrow can be congruent (points in the same direction as the flankers) or incongruent (points in the opposite direction to the flankers). The stimuli can appear above or below the fixation cross and can be cued with an asterisk (*) or not cued. There are three cue conditions: spatial cue (cue appears either above or below the fixation cross), centre cue (asterisk appears in the centre of the screen), or double cue (cue appears both above and below the fixation cross). Only the spatial cue indicates where the stimulus will appear, the location of the stimuli is ambiguous for the centre and double cue. Participants have a practice trial of 12 trials followed by three blocks of 96 trials (total of 288 trials). Feedback is only given during the practice trials. The ANT has been used in older adults to detect changes in attention compared to younger adults (Jennings et al., 2007).

RT is measured in milliseconds and a score is given for each of the domains. Alerting is measured by the no cue response time minus the double cue response time, for correct responses only. Orienting is measured by the centre cue response time minus the spatial cue response time, for correct responses only. Executive control is measured by response time in the incongruent flanker condition minus response time in the congruent condition, for correct responses only. High scores for alerting and orienting, and low scores on executive attention, indicate better performance. The task took approximately 10 min to complete.

#### Tip-of-the-tongue

Tip-of-the-tongue (TOT) instances were measured through a computerised task. In 60 trials, participants are shown a written definition of a word and are asked whether they can name the word the definition is referring to. There are three available responses: ‘Yes, I know the word’ (participant is given the answer and is asked whether that was the word they had in mind), ‘No, I don’t know the word’ (move onto next trial) or ‘tip-of-the-tongue’ (described to the participant as ‘you know the word but can’t bring it to mind just now, or you could think of it with more time’). In TOT instances, participants are then asked if they can recall how many syllables the word has, or if they can remember any of the sounds in the word. Responses are typed on the keyboard. Participants are then shown the answer and are asked whether that was the word they had in mind. A ‘Yes’ response at this stage is recorded as a true TOT instance, and a TOT score out of 60 is recorded for analysis. This TOT task has been used in previous research with older adults and was found to be related to cardiovascular health (Segaert et al., 2018). There were two 60-word lists for this task, which were counterbalanced across participants and sessions (pre- vs. post-intervention). The TOT task takes approximately 20 min.

### Procedure

All participants were screened on the inclusion and exclusion criteria and gave informed consent before being allocated to the intervention or active control group. Groups were allocated using stratified randomisation (split by sex), to ensure equal sex distribution in the intervention vs. control group, to avoid any potential confounds in performance on the tasks. Participants completed a baseline assessment of all outcome measures which took around two hours, as well as a questionnaire to record demographic information. Age, sex, ethnicity, highest educational qualification, and regular medication were all recorded. Cognitive measures were coded and administered using PsychoPy (Peirce et al., 2019).

The researcher then downloaded either the intervention application (Peak) or control application (Cardgames.io) onto the participant’s smartphone or tablet. Participants were shown how the applications work and were instructed to train for at least 15 min each day for the duration of the intervention period (three months). This training intensity and duration was based on a previous report which found that 50% of studies had less than 8 h and 20 min of training in total, and 90% of studies had less than 20 h of training (Noack et al., 2014). If adhered to correctly, our intervention would result in at least 22.5 h of training. While more training could in theory be more beneficial to our outcomes, we wanted to choose a duration and intensity that would be feasible and achievable for our chosen population. Adherence to the training programme was measured by asking participants to keep a training diary, which they reported through weekly emails detailing how much training they had completed that week (in minutes per day), what their Peak brain scores were (intervention) and what games they had played (active control). This also controlled for the amount of researcher contact both groups received.

Participants repeated the series of outcome measures immediately after the three-month intervention, to assess any direct effects of the training. At post-intervention, additional questions were asked about motivation (‘How motivated were you to complete the training?’) and enjoyment (‘How much did you enjoy the training?’) on a 7-point Likert scale from 1- ‘Not motivated’ / ‘I did not enjoy it’, to 7 – ‘Extremely motivated’ / ‘I really enjoyed it’. Adherence to the training was determined by asking participants how many days of training they missed per week.

The study was a randomised, controlled, single-blinded intervention: participants were randomly allocated and both groups were told the study aimed to investigate the effects of computerised smartphone apps on cognitive health, to ensure the same level of expectation effects (Kable et al., 2017). The study was approved by the University of Birmingham Ethics Committee (ERN_19-1176) and complied with ethical considerations outlined in the British Psychological Society Code of Human Research Ethics (BPS, 2021). Participants received monetary compensation for the in-person visits.

### Data reduction and analysis

Before analysis, data were cleaned to identify outliers, using Python 3.7. Outliers for RT data (for N-back, CRT, LC, and ANT tasks) were detected and removed from 2 ± SD from the mean, per participant per condition. This removed around 1% of RT responses. Individual responses were excluded from analysis if the % accuracy was below 50% for that task (this removed one participant’s response for CRT, four for the Letter Comparison task, four from the N-back task, four from the ANT, and two from the ToT task).

Pre- vs post-intervention cognitive scores were analysed in R Studio (RStudio Team, 2020). For those tasks where outcome variables had repeated observations over trials and/or participants (CRT, Letter Comparison task, and tip-of-the-tongue task), we ran linear mixed models using the lme4 (Bates et al., 2015) and report (Makowski et al., 2023) packages. Linear mixed models are extensions of regressions that are increasingly popular in psychological research (Bono et al., 2021) as a way of analysing data while being able to account for variability within factors such as item or participant (Judd et al., 2012). For each model, we attempted to include a maximal random-effects structure (Barr et al., 2013). We included participant and item as random intercepts (as there may be variance between items or individual differences in participants) and attempted to include as many random slopes (random adjustments to the fixed effects) as the model would allow. For other tasks, where the outcome measure is a single value (i.e., N-back d’, maximal digit span, or ANT cost score), linear regressions were conducted using the dplyr package (Wickham et al., 2023).

For the CRT task, 3-letter condition, and 6-letter condition of the Letter Comparison task, we fitted separate linear mixed models predicting reaction time, including session (pre vs post) and condition (intervention and control) as fixed effects. Random intercepts were included for participants and items. Random slopes for items were not included in the final models as they did not converge (Barr et al., 2013). These models were estimated using REML (Corbeil & Searle, 1976) and used nloptwrap optimizer. ToT data was analysed by a generalised linear mixed effects model, as the dependent variable (tip-of-the-tongue occurrence) was binomial (ToT response was either a 0 – no ToT, or 1 – ToT). ToT occurrence was predicted by fixed effects of session, condition, and the number of phonemes in each item (Segaert et al., 2018), as well as including random intercepts for item and participant. Random slopes were again not included in the final model due to non-convergence. ToT models were estimated using maximum likelihood and BOBYQA optimizer, with the logit link function. Maximal digit span, 1-back d’, 2-back d’, and alerting score, orienting score, and executive control score from the ANT were all investigated using session and condition as predictor variables in a linear regression model. As less than 5% of data was missing, missing data was not estimated (Jakobsen et al., 2017; Roth & Switzer III, 1995; Schafer, 1999). All continuous variables were scaled prior to analysis. As we were assessing many outcome variables and conducting multiple statistical analyses (*N* = 10), a Bonferroni adjusted p-value was calculated (new p-value = 0.005). 95% Confidence Intervals (CIs) and p-values were computed using a Wald t-distribution approximation.

## Results

### Demographics

Table 1 reports demographic information for the 103 participants who completed both pre- and post-intervention assessment sessions. There were no significant differences between groups at baseline in terms of age, gender, or MoCA score (all *p* >.05). Participants were excluded from further analyses if total training was less than 80% (less than 18 h in total during the three-month intervention). Four participants (*n* = 2 intervention, *n* = 2 control) were excluded due to low training, meaning a total of 99 participants (*n* = 50 intervention, *n* = 49 active control) were included in analysis. It is worth noting that, following Intent to Treat principles (Montori & Guyatt, 2001), we also ran analyses on the full dataset (*N* = 103). Overall effects did not change for any of the analyses reported below.

**Table 1:** Baseline demographics for intervention and active control groups

	Intervention (*n* = 52)	Active control (*n* = 51)	*p* value
**Age – years (SD)**	70.7 (5.5)	70.5 (5.6)	0.84
**MoCA score – M (SD)**	28.3 (1.3)	28.5 (1.5)	0.45
**Sex – N**			0.94
Female	31	30	
Male	19	19	
**Ethnicity – N**			0.99
White	48	47	
Mixed/Multiple Ethnic Groups	1	1	
Other	1	1	
**Highest educational qualification - N**			0.125
GCSEs, O-levels or equivalent	4	2	
A-levels or equivalent	7	15	
University undergraduate (e.g., BSc, BA)	13	14	
University postgraduate (e.g., MSc, MA, PhD)	12	13	
Other	13	5	

Note: MoCA = Montreal Cognitive Assessment

### Adherence to training and motivation

Overall adherence to both intervention and active control was high. Participants were asked to train for at least 15 min per day on their respective programmes for three months. The expected training time was a minimum of 22.5 h. On average, participants trained for 42.22 h (SD = 26.28, ranging from 18.67 to 176 h) and missed 4.5 days of training between baseline and follow-up during the three-month intervention. Total hours of training were calculated per participant by time trained across the three months, multiplying by the number of days participants trained for. Total training hours did not differ between intervention (M = 41.34, SD = 28.05) and active control (M = 43.13, SD = 24.59), t(97) = 0.336, *p* =.369.

Motivation to complete the training and enjoyment of the training were measured through questions post-intervention. There were no significant differences between intervention (M = 6.5, SD = 0.9) and active control (M = 6.3, SD = 0.7) in terms of motivation (*p* =.118) of the respective training programmes. There was also no significant difference between intervention (M = 6.3, SD = 1.1) and active control (M = 6.3, SD = 0.8) regarding training enjoyment (*p* =.895). Intervention and active control groups were therefore matched well in terms of motivation and enjoyment.

### Intervention group practice effects: performance on peak improves during the intervention

**Fig. 2:**
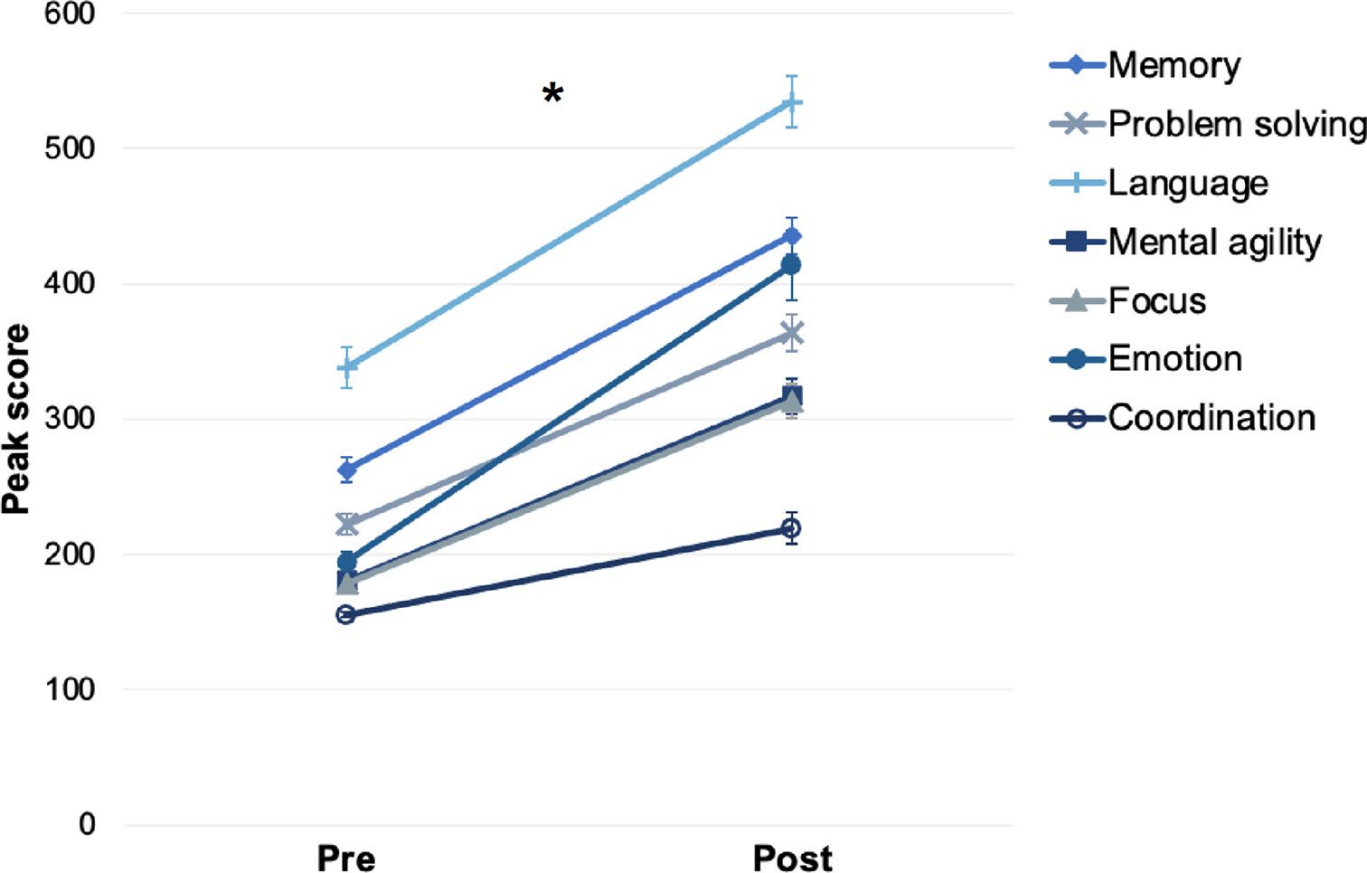
Pre- and post-intervention scores for Peak games, demonstrating practice effects within the intervention. Error bars reflect standard error. Peak scores are out of 1000

Repeated measures analyses of variance (ANOVAs) were conducted to compare first and last reported scores for the intervention group, for each of the seven Peak categories. Results were significant for all measures (all *p* <.001), indicating that participants in the intervention group improved significantly in their performance within the app (i.e. practice effects). Descriptives and results can be found in Table 2. Visual representations are shown in Fig. 2.

**Table 2:** Mean (SD) pre- and post-intervention peak scores for participants in the intervention group

Peak category	M (SD) pre-intervention (/1000)	M (SD) post-intervention (/1000)	ANOVA result (main effect of time)
Memory	262.47 (63.08)	435.49 (94.44)	F(1,50) = 206.08, *p* <.001, η^2^ = 0.81
Problem solving	222.12 (55.01)	363.51 (95.28)	F(1,50) = 121.77, *p* <.001, η^2^ = 0.71
Language	338.00 (107.48)	534.37 (133.22)	F(1,50) = 175.03, *p* <.001, η^2^ = 0.78
Mental agility	180.61 (29.63)	317.00 (93.55)	F(1,50) = 126.20, *p* <.001, η^2^ = 0.72
Focus	178.61 (26.38)	313.35 (89.67)	F(1,50) = 144.82, *p* <.001, η^2^ = 0.74
Emotion	194.41 (53.64)	413.45 (184.92)	F(1,50) = 98.56, *p* <.001, η^2^ = 0.66
Coordination	155.14 (14.34)	219.39 (85.02)	F(1,50) = 33.06, *p* <.001, η^2^ = 0.40

Note: Peak category scores are out of 1000

### Cognitive benefits of the intervention: pre- vs. post-intervention cognitive outcomes

**Fig. 3:**
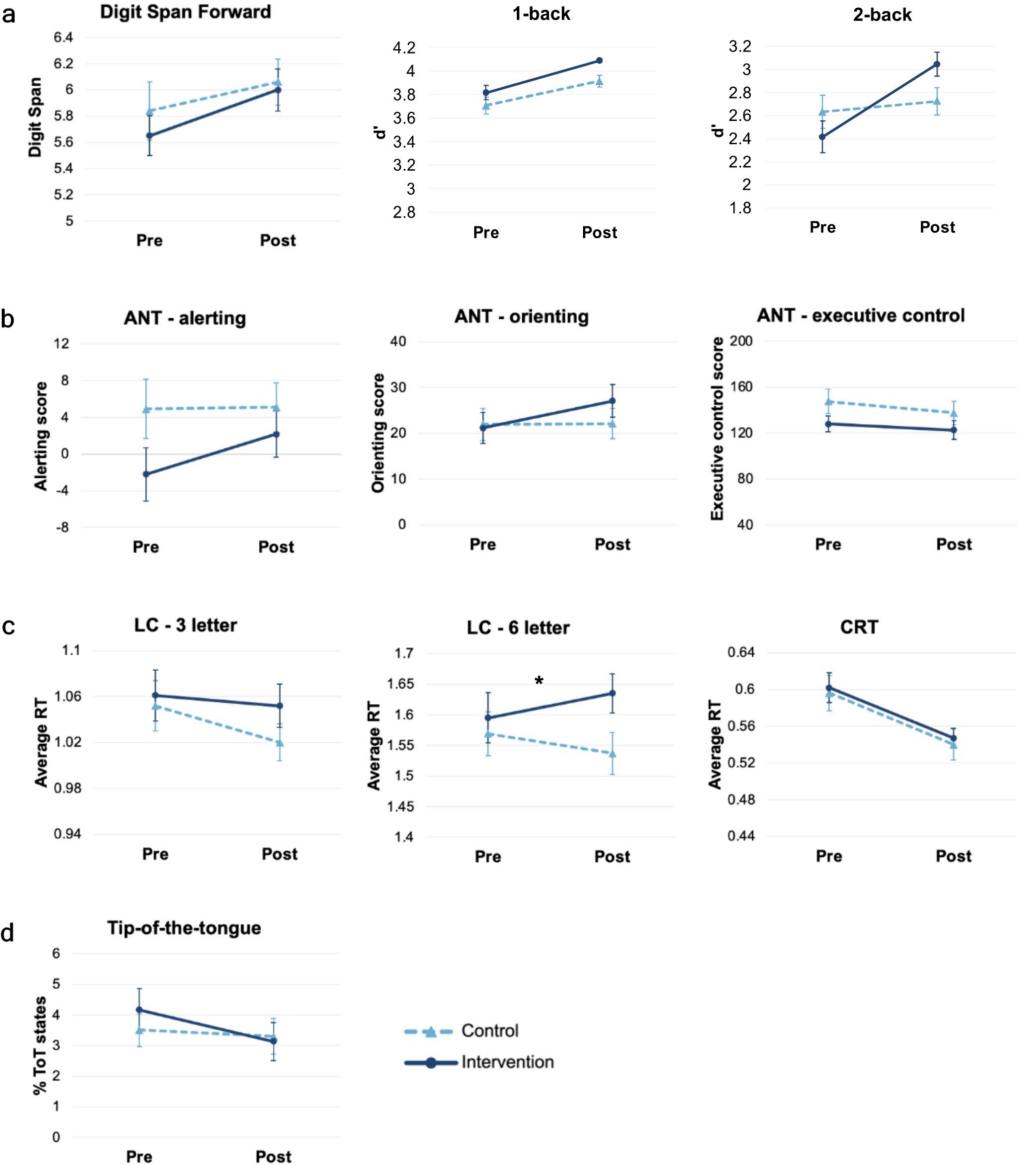
Pre- and post-intervention scores for outcome measures split by condition, demonstrating a lack of transfer effects of the intervention to the cognitive outcome measures. **a**: scores for working memory measures, analysed using linear regression. Higher scores represent better performance. **b**: scores for attention measures, analysed using linear regression. Higher performance for alerting and orienting, and lower scores for executive control, represent better performance. *c*: scores for processing speed measures, analysed using mixed models. Lower scores represent better performance. **d**: score for tip-of-the-tongue measure, analysed using mixed models. Lower scores represent better performance. Asterisks (*) represent significant session by condition interaction. Error bars reflect standard error

We investigated pre- versus post-intervention cognitive outcomes using mixed models (where there were repeated observations over participants and/or items) and linear regressions (where the outcome variable was one value) (see methods for more detailed descriptions). Raw pre- and post-intervention scores can be found in Table 3. Detail on best fitting models can be found in Table 4 (mixed models) and Table 5 (linear regressions). Visual representations can be found in Fig. 3. Alpha level (α) was adjusted for multiple comparisons (0.05 / 10 = 0.005).

Because dosage varied between participants, we also ran analyses to investigate whether dosage had any impact on pre- to post-intervention cognitive changes. Across all outcome measures, we only found one session (pre vs. post) by condition (training vs. control) by dosage (number of training hours) interaction, for the CRT (*p* =.001). However, the effect was in the opposite direction to what might be expected (i.e., those who trained for *less* time improved on their performance on the CRT in terms of response time at the second session). We do want to note also that we did not manipulate the dosage in this study, so there are certainly limitations to this additional (post-hoc) set of analyses. In light of this, we suspect that this one interaction with dosage is likely a spurious result.

**Table 3:** Pre-intervention and post-intervention scores in intervention and active control groups for cognitive outcome measures

Cognitive function	Measure	Group	Pre intervention [95%CI]	Post intervention [95%CI]
Working memory	Digit Span	Intervention	5.65 [5.27, 6.03]	6.00 [5.67, 6.33]
		Active control	5.84 [5.46, 6.22]	6.06 [5.73, 6.40]
	1back d’	Intervention	3.817 [3.69, 3.95]	3.914 [3.84, 3.99]
		Active control	3.706 [3.57, 3.84]	4.090 [4.02, 4.17]
	2back d’	Intervention	2.415 [2.14, 2.69]	3.045 [2.83, 3.26]
		Active control	2.632 [2.35, 2.91]	2.724 [2.50, 2.95]
Processing speed	CRT average RT (ms)	Intervention	0.602 [0.57, 0.64]	0.547 [0.52, 0.58]
		Active control	0.597 [0.56, 0.63]	0.540 [0.51, 0.57]
	LC 3 letter RT (ms)	Intervention	1.061 [1.02, 1.10]	1.052 [1.02, 1.09]
		Active control	1.052 [1.01, 1.10]	1.020 [0.99, 1.06]
	LC 6 letter RT (ms)	Intervention	1.595 [1.52, 1.67]	1.635 [1.57, 1.70]
		Active control	1.569 [1.49, 1.65]	1.537 [1.47, 1.60]
Attention	ANT alerting	Intervention	-2.19 [-8.27, 3.87]	2.18 [-2.96, 7.31]
		Active control	4.91 [-1.16, 10.98]	5.09 [-0.04, 10.23]
	ANT orienting	Intervention	21.14 [14.31, 27.97]	27.10 [20.25, 22.95]
		Active control	22.01 [15.17, 28.84]	22.12 [15.27, 28.97]
	ANT executive control (ms)	Intervention	128.00 [110.02, 145.98]	122.59 [104.24, 140.94]
		Active control	147.42 [129.44, 165.39]	137.44 [119.09, 155.97]
Language functioning	TOT states (%)	Intervention	4.17 [2.93, 5.40]	3.13 [1.93, 4.33]
		Active control	3.50 [2.25, 4.75]	3.30 [2.09, 4.51]

Note: CRT: Choice Reaction Time. LC: Letter Comparison. ANT: Attention Network Task. TOT: Tip-of-the-tongue. There were no significant differences between groups at baseline (all *p* >.1)

#### CRT task

We fitted a linear mixed model to predict reaction time from session (pre and post) and condition (intervention and active control). The model included item and participant ID as random effects. The total effect of both fixed and random factors (conditional R^2^) was 0.38, and the variance explained by the fixed effects alone (marginal R^2^) was 0.03. The main effect of session was significant (t(6058) = -10.63, *p* <.001), but we found no main effect of condition (*p* =.492) or interaction between session and condition (*p* =.907). Overall, RTs got faster post-intervention regardless of condition, after accounting for variability in items and participants.

#### Letter Comparison task

We fitted a linear mixed model to predict reaction time for the 3-letter condition of the Letter Comparison task from session (pre and post) and condition (intervention and active control). The model included item (each letter string) and participant ID as random effects. The variance of fixed and random factors together (conditional R^2^) was 0.42, and the variance explained by the fixed effects alone (marginal R^2^) was 0.003. The model showed a main effect of session (t(4399) = -4.29, *p* <.001) but no significant effect of condition (*p* =.917) or interaction between session and condition (*p* =.007), once Bonferroni adjusted p-values were accounted for. Similar to the CRT, after accounting for variance between item and participant, RTs got faster post-intervention, regardless of condition.

We fitted a linear mixed model to predict reaction time for the 6-letter condition of the Letter Comparison task from session (pre and post) and condition (intervention and active control). The model included item (each letter string) and participant ID as random effects. The variance of fixed and random factors together (conditional R^2^) was 0.45, and the variance explained by the fixed effects alone (marginal R^2^) was 0.005. After correcting for multiple comparisons, there was no significant effect of time (*p* =.01) or condition (*p* =.922) but there was a significant interaction between session and condition (t(3778) = 3.98, *p* <.001). Results showed that post (compared to pre) intervention, the control group significantly reduced their reaction times (i.e., they got faster), compared to the intervention group.

#### ToT

We fitted a logistic mixed model to predict ToT occurrences per trial with session, condition, and number of phonemes per item as fixed effects, as an increase in phonemes has been associated with increased ToT occurrences (Segaert et al., 2018). The model included item (each word) and participant ID as random effects. The variance of fixed and random factors together (conditional R^2^) was 0.33, and the variance explained by the fixed effects alone (marginal R^2^) was 0.01. After correcting for multiple comparisons, the model did not show significant effects of session (*p* =.373), condition (*p* =.356), number of phonemes (*p* =.05) or an interaction between session and condition (*p* =.006).

**Table 4:** Summary of the best fitting models for cognitive data analysed using linear mixed models

Task (outcome)	Predictor	Coefficient	95% CI	SE	t- or z-value	*p*
CRT task (RT)	Intercept	0.02	− 0.03, 0.07	0.028	0.731	0.497
	**Session**	**− 0.055**	**− 0.07**,** − 0.05**	**0.005**	**-10.63**	**< 0.001**
	Condition	0.013	− 0.02, 0.05	0.019	0.687	0.493
	Session*Condition	0.0008	− 0.01, 0.02	0.0007	0.117	0.907
Letter Comparison – 3 letter (RT)	Intercept	0.018	− 0.03, 0.06	0.023	0.776	0.439
	**Session**	**− 0.033**	**− 0.05**,** − 0.02**	**0.008**	**-4.295**	**< 0.001**
	Condition	− 0.003	− 0.06, 0.05	0.028	− 0.104	0.917
	Session*Condition	0.03	0.00, 0.05	0.011	2.72	0.007
Letter Comparison – 6 letter (RT)	Intercept	0.01	− 0.07, 0.09	0.043	0.224	0.824
	Session	− 0.035	− 0.06, − 0.00	0.014	-2.558	0.011
	Condition	0.004	− 0.08, 0.09	0.045	0.098	0.922
	Session*Condition	**0.077**	**0.04**,** 0.12**	**0.019**	**3.977**	**< 0.001**
ToT occurrence (0/1)	**Intercept**	**− 3.780**	**-4.15**,** -3.41**	**0.191**	**-19.787**	**< 0.001**
	Session	0.119	− 0.14, 0.38	0.134	0.891	0.373
	Condition	0.219	− 0.25, 0.69	0.238	0.922	0.356
	Nb Phonemes	0.098	0.00-0.20	0.050	1.960	0.050
	Session*Condition	-0.541	− 0.92, − 0.16	0.195	-2.770	0.006

Note. This analysis was conducted on *N* = 99 participants who completed more than 80% of minimum training hours. Bonferroni adjusted p-values were used based on *N* = 10 comparisons (*p* =.005). Significant effects that remained after correcting are highlighted in bold. T- values are used for CRT and Letter Comparison task. Z-value is used for ToT task

#### Working memory tasks

A linear regression predicting 1-back d’ was statistically significant and explained a moderate proportion of variance (F(3, 190) = 9.97, *p* <.001, adj. R^2^ = 0.12). There was a main effect of session (t(190) = 2.97, *p* =.003; Std. β = 0.57), but condition (*p* =.101) and interaction between session and condition (*p* =.658) were not significant. Results show that participants in both conditions improved performance in the post-intervention session compared to pre-intervention. It is important to note that scores here reflect a potential ceiling effect, as has been found in previous studies (e.g., Tusch et al., 2016).

A linear regression predicting 2-back d’ was statistically significant (F(3, 188) = 4.49, *p* =.005, adj. R^2^ = 0.05). However, after correcting for multiple comparisons (new *p* =.005), main effects of session (*p* =.739), condition (*p* =.232), and interaction between session and condition (*p* =.023) were not significant.

Linear regression predicting digit span from session (pre and post) and condition (intervention and control) was conducted. The model was not significant and explained little of the variance (F(3, 193) = 1.11, *p* =.344, adj. R^2^ = 0.002). There were no significant effects of session (*p* =.378), condition (*p* =.437) or an interaction between the two (*p* =.704). Participant performance did not change as a result of session or condition.

#### Attention Network Task

Similarly, linear regression predicting alerting score from session and condition was not significant (F(3, 191) = 1.23, *p* =.301, adj. R^2^ = 0.003). Results showed no significant effects of session (*p* =.843), condition (*p* =.083) or interaction between session and condition (*p* =.409). A linear regression predicting orienting score from session and condition was also not significant (F(3, 191) = 0.88, *p* =.455, adj. R^2^ = − 0.001). Results showed no significant effects of session (*p* =.96), condition (*p* =.861) or interaction between session and condition (*p* =.305). A final linear regression predicting executive control from session and condition was also not significant (F(3, 191) = 1.02, *p* =.383, adj. R^2^ = 0.0003). Results showed no significant effects of session (*p* =.493), condition (*p* =.149) or interaction between session and condition (*p* =.45). Results showed no effects of time or condition for any of the ANT cost scores.

**Table 5:** Summary of regression models for cognitive data analysed using linear regression

Outcome	Predictor	Coefficient	95% CI	SE	t-value	*p*
1-back d’	**Intercept**	**− 0.187**	**− 0.29**,** − 0.08**	**0.053**	**-3.505**	**< 0.001**
	**Session**	**0.226**	**0.08**,** 0.38**	**0.076**	**2.972**	**0.003**
	Condition	0.124	− 0.02, 0.27	0.075	1.650	0.101
	Session*Condition	0.047	− 0.16, 0.2	0.107	0.443	0.658
2-back d’	Intercept	− 0.067	− 0.32, 0.18	0.126	− 0.533	0.595
	Session	0.059	− 0.29, 0.41	0.178	0.333	0.739
	Condition	− 0.212	− 0.56, 0.14	0.177	-1.200	0.232
	Session*Condition	0.571	0.08, 1.06	0.250	2.286	0.023
Digit Span	Intercept	− 0.046	− 0.40, 0.31	0.180	− 0.259	0.796
	Session	0.224	− 0.28, 0.73	0.254	0.884	0.378
	Condition	− 0.197	− 0.70, 0.30	0.253	− 0.778	0.437
	Session*Condition	0.135	− 0.57, 0.84	0.358	0.378	0.706
ANT: Alerting score	Intercept	2.767	-2.92, 8.46	2.884	0.959	0.339
	Session	− 0.807	-8.81, 7.20	4.058	− 0.199	0.843
	Condition	-7.110	-15.16, 0.94	4.079	-1.743	0.083
	Session*Condition	4.741	-6.55, 16.03	5.725	0.828	0.409
ANT: Orienting score	Intercept	− 1.261	-8.12, 5.60	3.478	− 0.363	0.717
	Session	− 0.244	-9.90, 9.41	4.893	− 0.050	0.960
	Condition	− 0.868	-10.57, 8.83	4.918	− 0.176	0.860
	Session*Condition	7.104	-6.51, 20.72	6.902	1.029	0.305
ANT: executive control score	Intercept	12.259	-6.45, 30.97	9.486	1.292	0.198
	Session	-9.174	-35.50, 17.15	13.347	− 0.687	0.493
	Condition	-19.420	-45.88, 7.04	13.415	-1.448	0.149
	Session*Condition	8.419	-28.72, 45.56	18.828	0.447	0.655

Note. This analysis was conducted on *N* = 99 participants who completed more than 80% of minimum training hours. Bonferroni adjusted p-values were used based on *N* = 10 comparisons (*p* =.005). Significant effects that remained after correcting are highlighted in bold

## Discussion

This study explored whether cognitive training using Peak (a commercially available but relatively under-researched adaptive brain training programme) results in cognitive improvements in a sample of older adults. We designed the study in order to rigorously assess Peak: we used a randomised controlled intervention study design, with a sufficiently large sample of healthy older adults, a blinded active control group, and assessed the potential cognitive benefits using a comprehensive test battery (including multiple outcome measures for cognitive functions and including different cognitive domains). In line with our hypothesis, we found practice effects in the intervention group, specifically observing improvements on the Peak training game scores. However, we found no evidence of transfer to untrained tasks. While a subset of the previous research had suggested promising effects of brain training programmes (Anderson et al., 2013; Meltzer et al., 2023; Savulich et al., 2019), the literature is mixed and many other studies, alongside our own, find little to no transfer effects (Guye & von Bastian, 2017; Stojanoski et al., 2018).

Our data demonstrated that participants in our intervention condition significantly improved their game scores for all seven of Peak’s categories: Memory, Problem Solving, Language, Mental Agility, Focus, Emotion, and Coordination. Such practice effects are shown regularly in brain training studies, so this was not unexpected. We also found some test re-test effects, where both our intervention and active control group improved in performance on our cognitive outcome measures. Test re-test effects (which are well documented in the literature (Scharfen et al., 2018) as well as improvements within the training programme are unlikely to indicate true improvements in cognitive abilities as a result of the intervention. Instead, they are merely learning effects of being able to do the training task.

The key aim for the present study was to assess whether brain training leads to transferable benefits to wider cognitive abilities. We assessed cognitive performance pre- and post-brain training intervention, using multiple measures of attention, working memory, processing speed, and language functioning. We did not find any evidence that brain training significantly improved cognitive performance in the intervention group when comparing to the active control. As mentioned in the introduction, transfer effects are few and far between in existing brain training literature, and it is possible that ‘transfer effects’ in published research are sometimes misinterpreted practice effects instead. For example, one recent study reported transfer effects for a composite of memory measures, however these were driven solely by improvements in N-back training (Lee et al., 2020). Upon closer examination of Lee et al., 2020, the cognitive training programme administered in the intervention group (BrainHQ) involved an N-back style game, and therefore any suggested transfer is arguably due to a practice effect.

It is important in this field to closely scrutinise previous research, as the terms practice effects and transfer effects are sometimes not clearly defined or fully explained. For example, one recent study analysed retrospective data from Peak and suggested that from a sample of 12,000 users, processing speed increased after 100 sessions of Peak training (Bonnechere et al., 2021). While this finding is impressive at face value, what is important here is that the authors’ measure of processing speed was performance on the trained games and thus should be interpreted as practice effects. Our data show practice benefits do indeed take place during brain training, but in the present study at least, this does not transfer to other measures of the same cognitive constructs, or to different cognitive constructs.

The only session by condition interaction we found was for the 6-letter condition of the Letter Comparison task, where data showed that reaction times were faster in the active control group compared to the intervention group post vs. pre-intervention. There was a similar trend for the 3-letter condition. These findings are slightly unexpected, but the finding that a control group improves more than a cognitive training group has previously been shown (Hardy et al., 2015; van Muijden et al., 2012). Further, the effect is small, with the average RT in the intervention group increasing by 40ms, while the control group decreasing by 32ms. We believe this interaction is a spurious one, as the games the control group played do not have any features that may have indirectly trained processing speed (e.g., time limits), though we cannot rule out the possibility that some aspect of the control condition may have had this effect.

There are potential issues with our choice of active control; it is a similar-form control rather than an active-ingredient control (Masurovsky, 2020). However, our active control group, who played computer games on a free smartphone application, was similar regarding the novelty and variety of tasks. We found no significant differences between our intervention and active control groups in terms of motivation or enjoyment. This is important as research has shown these factors can affect intervention success (Green & Bavelier, 2008). Furthermore, there were no significant differences between groups in how much time participants trained for. This shows that the active control group was a suitable match for the experimental condition – they were comparable in these important factors, but their training did not include the brain training element. Note that our study design is comparable to a cognitive training study that used Lumosity to assess improvements in executive function in younger adults (Kable et al., 2017). Similar to our study, they used an active control group that was matched to the intervention group in terms of engagement, motivation and novelty (Kable et al., 2017). In line with our findings, they found practice effects but did not find any evidence of transfer effects from the brain training programme to any cognitive outcome measures. We have both corroborated the findings from this study and extended the research to an older adult population.

Although our data suggest there are no direct benefits of brain training on cognitive performance in the older adult population, there may be indirect benefits. There is rarely a negative impact of using these programmes, and perhaps a belief of these applications working might lead to improvements in wellbeing, which itself is helpful. For example, worries about cognitive health has been associated with poorer psychological wellbeing (Sutton et al., 2022) and have even been linked with poorer cognitive performance (Caughie et al., 2021). Therefore, even if these applications do not directly improve general cognitive ability, if they reduce worry about cognitive decline in older adults this is still beneficial. This is a possible avenue for future research.

Limitations of the present study include that the sample consisted of mainly White participants, which limits the generalisability of the findings. A positive of our sample however is that the sample size was larger than 90% of previous cognitive training studies (Noack et al., 2014). While our intervention was adaptive, i.e. it increased with difficulty as participant performance improves, it would have been beneficial to include an explicit measure to assess whether the intervention sufficiently challenged participants, or whether there were differences between conditions, as per Lövdén et al.’s (2010) framework of cognitive plasticity. We did not include a passive control group, which would have allowed direct comparison of training effects without revealing to the active control group what condition they were in. Timescales and study design did not allow for this in the present study, but this could be beneficial in future research. We acknowledge that only one language functioning task was included in the methodology, however, language functioning is time-consuming to measure. Including multiple measures of language in future research (such as measures of comprehension or sentence production, e.g. (Fernandes et al., 2024) would be valuable. Finally, one could argue that the flexibility participants were afforded in training duration is a limitation of our study design. Indeed, there were large differences in how long participants trained for; some maintained the minimum 15 min a day, but many went above and beyond. While we designed this intervention to be controlled and robust, we also wanted it to be enjoyable and not too restrictive for the participants. A small pilot study found limiting training to 15 min a day was difficult due to participants enjoyment of the training, so we removed this requirement for the study presented here. Importantly, training duration did not significantly differ between the intervention and the control group, so this is unlikely to have impacted our findings.

In sum, we have shown that making a clear distinction between transfer and practice effects in the cognitive training literature is important. A recent meta-analysis concluded that at present there is no convincing empirical evidence to suggest brain training programmes lead to tangible transfer effects in older adults (Nguyen et al., 2022). Our data is in line with this and suggests that commercial brain training leads to practice effects, without convincing evidence of transfer to cognitive abilities beyond the practiced tasks. In short, we offer a rigorous investigation into a brain training product (i.e. Peak) which had not been studied extensively, and in our sample of healthy older adults, practice makes perfect, but it does not transfer to wider cognitive benefits.

## Data Availability

The data generated during and/or analysed during the current study are available on our OSF project: 10.17605/OSF.IO/W46XU.

## References

[CR1] Anderson, S., White-Schwoch, T., Parbery-Clark, A., & Kraus, N. (2013). Reversal of age-related neural timing delays with training. *Proceedings of the National Academy of Sciences of the United States of America*, *110*(11), 4357–4362. 10.1073/pnas.1213555110 23401541 10.1073/pnas.1213555110 PMC3600492

[CR2] Ball, K., Berch, D. B., Helmers, K. F., Jobe, J. B., Leveck, M. D., Marsiske, M., Morris, J. N., Rebok, G. W., Smith, D. M., Tennstedt, S. L., Unverzagt, F. W., & Willis, S. L. (2002). Effects of cognitive training interventions with older adults A randomized controlled trial. *Journal of the American Medical Association*, *288*(18), 2271–2281. https://jamanetwork.com/journals/jama/articlepdf/195506/joc21020.pdf 12425704 10.1001/jama.288.18.2271 PMC2916176

[CR3] Ballesteros, S., Mayas, J., Prieto, A., Ruiz-Marquez, E., Toril, P., & Reales, J. M. (2017). Effects of video game training on measures of selective attention and working memory in older adults: Results from a randomized controlled trial. *Frontiers in Aging Neuroscience*, *9*, 354. 29163136 10.3389/fnagi.2017.00354 PMC5671951

[CR4] Barr, D. J., Levy, R., Scheepers, C., & Tily, H. J. (2013). Random effects structure for confirmatory hypothesis testing: Keep it maximal. *Journal of Memory and Language*, *68*(3), 255–278. 10.1016/j.jml.2012.11.001 PMC3881361 24403724

[CR5] Bates, D., Mächler, M., Bolker, B., & Walker, S. (2015). Fitting linear mixed-effects models Usinglme4. *Journal of Statistical Software*, *67*(1). 10.18637/jss.v067.i01

[CR7] Bonnechere, B., Langley, C., & Sahakian, B. J. (2020). The use of commercial computerised cognitive games in older adults: A meta-analysis. *Scientific Reports*, *10*(1), 15276. 10.1038/s41598-020-72281-3 32943742 10.1038/s41598-020-72281-3 PMC7498601

[CR6] Bonnechere, B., Klass, M., Langley, C., & Sahakian, B. J. (2021). Brain training using cognitive apps can improve cognitive performance and processing speed in older adults. *Scientific Reports*, *11*(1), 12313. 10.1038/s41598-021-91867-z 34112925 10.1038/s41598-021-91867-z PMC8192763

[CR8] Bono, R., Alarcon, R., & Blanca, M. J. (2021). Report quality of generalized linear mixed models in psychology: A systematic review. *Frontiers in Psychology*, *12*, 666182. 10.3389/fpsyg.2021.666182 33967923 10.3389/fpsyg.2021.666182 PMC8100208

[CR9] BPS, B. P. S. (2021). *BPS code of human research ethics*.

[CR10] BrainHQ (2023). Retrieved 21/02/23 from https://www.brainhq.com/

[CR11] Brehmer, Y., Westerberg, H., & Bäckman, L. (2012). Working-memory training in younger and older adults: Training gains, transfer, and maintenance. *Frontiers in Human Neuroscience*, *MARCH 2012*. 10.3389/fnhum.2012.00063 10.3389/fnhum.2012.00063 PMC3313479 22470330

[CR12] Cardgames.io (2023). Retrieved 23/02/23 from https://cardgames.io

[CR13] Carson, N., Leach, L., & Murphy, K. J. (2018). A re-examination of Montreal cognitive assessment (MoCA) cutoff scores. *International Journal of Geriatric Psychiatry*, *33*(2), 379–388. 10.1002/gps.4756 28731508 10.1002/gps.4756

[CR14] Caughie, C., Bean, P., Tiede, P., Cobb, J., McFarland, C., & Hall, S. (2021). Dementia worry and neuropsychological performance in healthy older adults. *Archives of Clinical Neuropsychology: the official Journal of the National Academy of Neuropsychologists*, *36*(1), 29–36. 10.1093/arclin/acaa057 32793959 10.1093/arclin/acaa057

[CR15] Corbeil, R. R., & Searle, S. R. (1976). Restricted maximum likelihood (REML) Estimation of variance components in the mixed model. *Technometrics*, *18*(1). 10.2307/1267913

[CR16] Deary, I. J., Liewald, D., & Nissan, J. (2010). A free, easy-to-use, computer-based simple and four-choice reaction time programme: The Deary-Liewald reaction time task. *Behavior Research Methods*, *43*(1), 258–268. 10.3758/s13428-010-0024-1 10.3758/s13428-010-0024-1 21287123

[CR17] Eriksen, B. A., & Eriksen, C. W. (1974). Effects of noise letters upon the identification of a target letter in a nonsearch task. *Perception & Psychophysics*, *16*(1), 143–149. 10.3758/bf03203267

[CR80] Feier, C. D., & Gerstman, L. J. (1980). Sentence comprehension abilities throughout the adult life span. *Journal of Gerontology*, *35*(5), 722–728. 10.1093/geronj/35.5.722 7430569

[CR18] Fernandes, E. G., Segaert, K., Rahman, F., Wetterlin, A., & Wheeldon, L. (2024). Bilingualism and ageing independently impact on Language processing: Evidence from comprehension and production. *Bilingualism: Language and Cognition*, 1–15.

[CR19] Glisky, E. L. (2007). Changes in cognitive function in human aging. *Brain Aging*, 3–20. 21204355

[CR20] Green, C. S., & Bavelier, D. (2008). Exercising your brain: A review of human brain plasticity and training-induced learning. *Psychology and Aging*, *23*(4), 692–701. 10.1037/a0014345 19140641 10.1037/a0014345 PMC2896818

[CR21] Green, C. S., & Bavelier, D. (2012). Learning, attentional control, and action video games. *Current Biology*, *22*(6), R197–206. 10.1016/j.cub.2012.02.012 22440805 10.1016/j.cub.2012.02.012 PMC3461277

[CR22] Green, C. S., Strobach, T., & Schubert, T. (2014). On methodological standards in training and transfer experiments. *Psychological Research Psychologische Forschung*, *78*(6), 756–772. 10.1007/s00426-013-0535-3 24346424 10.1007/s00426-013-0535-3

[CR23] Grégoire, J., & Van der Linden, M. (1997). Effect of age on forward and backward digit spans. *Aging Neuropsychology and Cognition*, *4*(2), 140–149.

[CR24] Guye, S., & von Bastian, C. C. (2017). Working memory training in older adults: Bayesian evidence supporting the absence of transfer. *Psychology and Aging*, *32*(8). 10.1037/pag0000206 10.1037/pag0000206 29239658

[CR25] Hardy, J. L., Nelson, R. A., Thomason, M. E., Sternberg, D. A., Katovich, K., Farzin, F., & Scanlon, M. (2015). Enhancing cognitive abilities with comprehensive training: A large, online, randomized, active-controlled trial. *Plos One*, *10*(9), e0134467. 10.1371/journal.pone.0134467 26333022 10.1371/journal.pone.0134467 PMC4557999

[CR26] Jaeggi, S. M., Buschkuehl, M., Perrig, W. J., & Meier, B. (2010). The concurrent validity of the N-back task as a working memory measure. *Memory (Hove, England)*, *18*(4), 394–412. 10.1080/09658211003702171 20408039 10.1080/09658211003702171

[CR27] Jakobsen, J. C., Gluud, C., Wetterslev, J., & Winkel, P. (2017). When and how should multiple imputation be used for handling missing data in randomised clinical trials–a practical guide with flowcharts. *BMC Medical Research Methodology*, *17*(1), 1–10. 29207961 10.1186/s12874-017-0442-1 PMC5717805

[CR28] Jennings, J. M., Dagenbach, D., Engle, C. M., & Funke, L. J. (2007). Age-related changes And the attention network task: An examination of alerting, orienting, And executive function. *Neuropsychology, Development, and Cognition. Section B, Aging, Neuropsychology and Cognition*, *14*(4), 353–369. 10.1080/13825580600788837 17612813 10.1080/13825580600788837

[CR29] Judd, C. M., Westfall, J., & Kenny, D. A. (2012). Treating stimuli as A random factor in social psychology: A new and comprehensive solution to A pervasive but largely ignored problem. *Journal of Personality and Social Psychology*, *103*(1), 54. 22612667 10.1037/a0028347

[CR30] Kable, J. W., Caulfield, M. K., Falcone, M., McConnell, M., Bernardo, L., Parthasarathi, T., Cooper, N., Ashare, R., Audrain-McGovern, J., Hornik, R., Diefenbach, P., Lee, F. J., & Lerman, C. (2017). No effect of commercial cognitive training on brain activity, choice behavior, or cognitive performance. *Journal of Neuroscience*, *37*(31), 7390–7402. 10.1523/JNEUROSCI.2832-16.2017 28694338 10.1523/JNEUROSCI.2832-16.2017 PMC5546110

[CR31] Karbach, J., & Verhaeghen, P. (2014). Making working memory work: A Meta-Analysis of Executive-Control and working memory training in older adults. *Psychological Science*, *25*(11), 2027–2037. 10.1177/0956797614548725 25298292 10.1177/0956797614548725 PMC4381540

[CR32] Kueider, A. M., Parisi, J. M., Gross, A. L., & Rebok, G. W. (2012). Computerized cognitive training with older adults: A systematic review. *Plos One*, *7*(7), e40588. 10.1371/journal.pone.0040588 22792378 10.1371/journal.pone.0040588 PMC3394709

[CR33] Lampit, A., Hallock, H., & Valenzuela, M. (2014). Computerized cognitive training in cognitively healthy older adults: A systematic review and Meta-Analysis of effect modifiers. *PLoS Medicine*, *11*(11). 10.1371/journal.pmed.1001756 10.1371/journal.pmed.1001756 PMC4236015 25405755

[CR34] Lee, H. K., Kent, J. D., Wendel, C., Wolinsky, F. D., Foster, E. D., Merzenich, M. M., & Voss, M. W. (2020). Home-Based, adaptive cognitive training for cognitively normal older adults: Initial efficacy trial. *Journals of Gerontology. Series B, Psychological Sciences and Social Sciences*, *75*(6), 1144–1154. 10.1093/geronb/gbz073 31140569 10.1093/geronb/gbz073 PMC7265807

[CR35] Lumosity (2023). Retrieved 21/02/23 from https://www.lumosity.com/en/

[CR36] Makowski, D., Lüdecke, D., Patil, I., Thériault, R., Ben-Shachar, M. S., & Wiernik, B. M. (2023). *Automated results reporting as a practical tool to improve reproducibility and methodological best practices adoption. CRAN.* https://easystats.github.io/report/

[CR37] Masurovsky, A. (2020). Controlling for placebo effects in computerized cognitive training studies with healthy older adults from 2016–2018: Systematic review. *JMIR Serious Games*, *8*(2), e14030. 10.2196/14030 32589159 10.2196/14030 PMC7381254

[CR38] Maylor, E. A. (1990). Age, blocking and the tip of the tongue state. *British Journal of Psychology*, *81*(2), 123–134. 2364243 10.1111/j.2044-8295.1990.tb02350.x

[CR39] McDougall, S., & House, B. (2012). Brain training in older adults: Evidence of transfer to memory span performance and pseudo-Matthew effects. *Neuropsychology, Development, and Cognition. Section B, Aging, Neuropsychology and Cognition*, *19*(1–2), 195–221. 10.1080/13825585.2011.640656 22248429 10.1080/13825585.2011.640656

[CR40] Meltzer, J. A., Kates Rose, M., Le, A. Y., Spencer, K. A., Goldstein, L., Gubanova, A., Lai, A. C., Yossofzai, M., Armstrong, S. E. M., & Bialystok, E. (2023). Improvement in executive function for older adults through smartphone apps: A randomized clinical trial comparing Language learning and brain training. *Neuropsychology, Development, and Cognition. Section B, Aging, Neuropsychology and Cognition*, *30*(2), 150–171. 10.1080/13825585.2021.1991262 34694201 10.1080/13825585.2021.1991262

[CR41] Montori, V. M., & Guyatt, G. H. (2001). Intention-to-treat principle. *Cmaj*, *165*(10), 1339–1341. 11760981 PMC81628

[CR42] Motter, J. N., Grinberg, A., Lieberman, D. H., Iqnaibi, W. B., & Sneed, J. R. (2019). Computerized cognitive training in young adults with depressive symptoms: Effects on mood, cognition, and everyday functioning. *Journal of Affective Disorders*, *245*, 28–37. 10.1016/j.jad.2018.10.109 30366235 10.1016/j.jad.2018.10.109

[CR43] Nasreddine, Z. S., Phillips, N. A., Bédirian, V., Charbonneau, S., Whitehead, V., Collin, I., Cummings, J. L., & Chertkow, H. (2005). The Montreal cognitive assessment, MoCA: A brief screening tool for mild cognitive impairment. *Journal of the American Geriatrics Society*, *53*(4), 695–699.10.1111/j.1532-5415.2005.53221.x. 15817019 10.1111/j.1532-5415.2005.53221.x

[CR45] Nguyen, L., Murphy, K., & Andrews, G. (2022). A game a day keeps cognitive decline away?? A systematic review and Meta-Analysis of Commercially-Available brain training programs in healthy and cognitively impaired older adults. *Neuropsychology Review*, *32*(3), 601–630. 10.1007/s11065-021-09515-2 34251578 10.1007/s11065-021-09515-2

[CR46] Noack, H., Lövdén, M., & Schmiedek, F. (2014). On the validity and generality of transfer effects in cognitive training research. *Psychological Research Psychologische Forschung*, *78*(6), 773–789. 10.1007/s00426-014-0564-6 24691586 10.1007/s00426-014-0564-6

[CR47] Peak (2023). Retrieved 21/02/23 from https://peak.net

[CR48] Peirce, J., Gray, J. R., Simpson, S., MacAskill, M., Hochenberger, R., Sogo, H., Kastman, E., & Lindelov, J. K. (2019). PsychoPy2: Experiments in behavior made easy. *Behavior Research Methods*, *51*(1), 195–203. 10.3758/s13428-018-01193-y 30734206 10.3758/s13428-018-01193-y PMC6420413

[CR49] Posner, M. I., & Cohen, Y. (1984). Components of visual orienting. *Attention and Performance X: Control of Language Processes*, *32*, 531–556.

[CR50] Rabipour, S., & Davidson, P. S. R. (2015). Do you believe in brain training? A questionnaire about expectations of computerised cognitive training. *Behavioural Brain Research*, *295*, 64–70. 10.1016/j.bbr.2015.01.002 25591472 10.1016/j.bbr.2015.01.002

[CR51] Rebok, G. W., Ball, K., Guey, L. T., Jones, R. N., Kim, H. Y., King, J. W., Marsiske, M., Morris, J. N., Tennstedt, S. L., Unverzagt, F. W., & Willis, S. L. (2014). Ten-year effects of the advanced cognitive training for independent and vital elderly cognitive training trial on cognition and everyday functioning in older adults. *Journal of the American Geriatrics Society*, *62*(1), 16–24. 10.1111/jgs.12607 24417410 10.1111/jgs.12607 PMC4055506

[CR52] Reuter-Lorenz, P. A., Festini, S. B., & Jantz, T. K. (2021). Executive functions and neurocognitive aging. *Handbook of the psychology of aging* (pp. 67–81). Elsevier.

[CR53] Roser, M., Ortiz-Ospina, E., & Ritchie, H. (2013). *Life Expectancy*. Retrieved 19th February 2021 from ‘https://ourworldindata.org/life-expectancy [Online Resource].

[CR54] Roth, P. L., & Switzer, I. I. I., F. S (1995). A Monte Carlo analysis of missing data techniques in a HRM setting. *Journal of Management*, *21*(5), 1003–1023.

[CR55] RStudio Team, R. (2020). *RStudio: Integrated Development for R.* In RStudio, PBC, Boston, MA. http://www.rstudio.com/

[CR56] Sala, G., Aksayli, N. D., Tatlidil, K. S., Tatsumi, T., Gondo, Y., Gobet, F., Zwaan, R., & Verkoeijen, P. (2019). Near and Far transfer in cognitive training: A Second-Order Meta-Analysis. *Collabra: Psychology*, *5*(1). 10.1525/collabra.203

[CR57] Salthouse, T. A. (2010). Selective review of cognitive aging. *Journal of the International Neuropsychological Society*, *16*(5), 754–760. 10.1017/S1355617710000706 20673381 10.1017/S1355617710000706 PMC3637655

[CR58] Salthouse, T. A., & Babcock, R. L. (1991). Decomposing adult age differences in working memory. *Developmental Psychology*, *27*(5), 763–776. 10.1037/0012-1649.27.5.763

[CR59] Sandberg, P., Rönnlund, M., Nyberg, L., & Stigsdotter Neely, A. (2014). Executive process training in young and old adults. *Aging Neuropsychology and Cognition*, *21*(5), 577–605. 10.1080/13825585.2013.839777 10.1080/13825585.2013.839777 24148093

[CR60] Savulich, G., Thorp, E., Piercy, T., Peterson, K. A., Pickard, J. D., & Sahakian, B. J. (2019). Improvements in attention following cognitive training with the novel decoder game on an ipad. *Frontiers in Behavioral Neuroscience*, 13. 10.3389/fnbeh.2019.00002 10.3389/fnbeh.2019.00002 PMC6348266 30719000

[CR61] Schafer, J. L. (1999). Multiple imputation: A primer. *Statistical Methods in Medical Research*, *8*(1), 3–15. 10347857 10.1177/096228029900800102

[CR62] Scharfen, J., Peters, J. M., & Holling, H. (2018). Retest effects in cognitive ability tests: A meta-analysis. *Intelligence*, *67*, 44–66. 10.1016/j.intell.2018.01.003

[CR63] Segaert, K., Lucas, S. J. E., Burley, C. V., Segaert, P., Milner, A. E., Ryan, M., & Wheeldon, L. (2018). Higher physical fitness levels are associated with less Language decline in healthy ageing. *Scientific Reports*, *8*(1). 10.1038/s41598-018-24972-1 10.1038/s41598-018-24972-1 PMC5928071 29712942

[CR64] Simons, D. J., Boot, W. R., Charness, N., Gathercole, S. E., Chabris, C. F., Hambrick, D. Z., & Stine-Morrow, E. A. (2016). Do Brain-Training programs work?? *Psychological Science in the Public Interest,*, *17*(3), 103–186. 10.1177/1529100616661983 27697851 10.1177/1529100616661983

[CR65] Stojanoski, B., Lyons, K. M., Pearce, A. A. A., & Owen, A. M. (2018). Targeted training: Converging evidence against the transferable benefits of online brain training on cognitive function. *Neuropsychologia*, *117*, 541–550. 10.1016/j.neuropsychologia.2018.07.013 30009838 10.1016/j.neuropsychologia.2018.07.013

[CR66] Sutton, E., Catling, J., Segaert, K., & van Veldhuijzen, J. (2022). Cognitive health worries, reduced physical activity and fewer social interactions negatively impact psychological wellbeing in older adults during the COVID-19 pandemic. *Frontiers in Psychology*, *13*, 823089. 10.3389/fpsyg.2022.823089 35250763 10.3389/fpsyg.2022.823089 PMC8891508

[CR67] Tetlow, A. M., & Edwards, J. D. (2017). Systematic literature review and Meta-Analysis of commercially available computerized cognitive training among older adults. *Journal of Cognitive Enhancement*, *1*(4), 559–575. 10.1007/s41465-017-0051-2

[CR68] Tusch, E. S., Alperin, B. R., Ryan, E., Holcomb, P. J., Mohammed, A. H., & Daffner, K. R. (2016). Changes in neural activity underlying working memory after computerized cognitive training in older adults. *Frontiers in Aging Neuroscience*, *8*, 255. 27877122 10.3389/fnagi.2016.00255 PMC5099139

[CR69] van Muijden, J., Band, G. P. H., & Hommel, B. (2012). Online games training aging brains: Limited transfer to cognitive control functions. *Frontiers in Human Neuroscience(JULY)*. 10.3389/fnhum.2012.00221 10.3389/fnhum.2012.00221 PMC3421963 22912609

[CR70] Veríssimo, J., Verhaeghen, P., Goldman, N., Weinstein, M., & Ullman, M. T. (2022). Evidence that ageing yields improvements as well as declines across attention and executive functions. *Nature Human Behaviour*, *6*(1), 97–110. 34413509 10.1038/s41562-021-01169-7

[CR71] Wickham, H., François, R., Henry, L., Müller, K., & Vaughan, D. (2023). *dplyr: A Grammar of Data Manipulation.* https://dplyr.tidyverse.org, https://github.com/tidyverse/dplyr

